# Antimicrobial resistance in wastewater-impacted coastal waters: implications for environmental surveillance and public health

**DOI:** 10.1099/mic.0.001771

**Published:** 2026-09-07

**Authors:** Niamh Cahill, Aneta Kovarova, Zina Alfahl, Louise O’Connor, Dearbháile Morris

**Affiliations:** 1 Antimicrobial and Microbial Ecology Group, School of Medicine, University of Galway, Galway, Ireland; 2 Centre for One Health, Ryan Institute, University of Galway, Galway, Ireland; 3 Molecular Diagnostics Research Group, School of Biological and Chemical Sciences, University of Galway, Galway, Ireland

**Keywords:** antimicrobial resistance, environmental surveillance, *Escherichia coli*, One Health, surface waters, wastewater

## Abstract

Antimicrobial resistance (AMR) is a global public health concern, and wastewater-impacted aquatic environments are recognized as reservoirs and dissemination pathways for antimicrobial-resistant bacteria (ARB) and genes (ARGs). However, harmonized environmental AMR surveillance frameworks that integrate culture-based ARB enumeration with molecular ARG monitoring across wastewater and receiving surface waters remain limited. This pilot study, conducted in Ireland as part of a European harmonized monitoring initiative, assessed ARB and ARG abundances in wastewaters and surface waters. Wastewater treatment plant influent (*n*=3), effluent (*n*=3) and surface waters upstream and downstream of the discharge point (*n*=3 each) were sampled on three occasions in late 2024. Culture-based methods enumerated total and extended-spectrum *β*-lactamase (ESBL)-producing *Escherichia coli*, while quantitative real-time PCR quantified 16S rRNA and five AMR-associated genes (*intI1*, *ermB*, *aadA1*, *bla*_CTX-M-1_ and *vanA*). Total *E. coli* concentrations were highest in influent (~10^5^–10^6^ c.f.u. dl^−1^), decreased in effluent (~10^3^–10^5^ c.f.u. dl^−1^) and lowest in surface waters (~10¹−10² c.f.u. dl^−1^). ESBL-producing *E. coli* were consistently detected in influent (~10^4^ c.f.u. dl^−1^) and effluent (~10^1^–10^3^ c.f.u. dl^−1^) but were not recovered from seawater. ARG abundances were highest in influent, reaching up to ~10^11^ copies dl^−1^ for *intl1*, remained elevated in effluent (up to ~10^10^ copies dl^−1^) and were ~2–3 orders of magnitude lower in surface waters relative to effluent. Downstream seawater exhibited higher ARG levels than upstream freshwater despite low culturable *E. coli*. Peak ARG concentrations in effluent and surface waters were observed following a period of heavy rainfall; however, the limited number of sampling events precluded assessment of any statistical association. These findings highlight the impact of wastewater discharges on environmental AMR dissemination and suggest that faecal indicator-based monitoring may underestimate emerging risks, supporting integration of AMR indicators into EU water quality frameworks.

## Graphical abstract

Overview of antimicrobial resistance across wastewater and receiving waters, illustrating changes in bacterial load and antimicrobial-resistant bacteria and gene abundance from upstream freshwater to downstream seawater.

## Introduction

Antimicrobial resistance (AMR) is recognized as one of the most concerning global public health threats at present. Bacterial AMR alone was estimated to have directly caused ~1.14 million deaths globally in 2021 and, without coordinated global action, is projected to increase to an estimated 1.91 million deaths annually by 2050, posing significant socioeconomic and health consequences [1].

The World Health Organization (WHO) Global Action Plan on AMR highlights the need for a One Health approach, recognizing the interconnected roles of human, animal and environmental health in the emergence and spread of resistance [2].

Despite advances in clinical and veterinary AMR surveillance, routine environmental AMR surveillance, particularly in aquatic ecosystems, remains limited and lacks harmonized monitoring approaches [3, 4]. Considering the projected increase in AMR-related mortality, understanding environmental reservoirs is crucial for the development of effective interventions. The WHO Global Call to Action on AMR (2025) reinforces the need for coordinated cross-sectoral action, harmonized AMR data collection and efforts to reduce antimicrobials entering the environment [5].

Aquatic environments are increasingly recognized as key reservoirs and dissemination pathways for antimicrobial-resistant bacteria (ARB) and genes (ARG), receiving inputs from diverse sources such as municipal wastewater, hospital effluents, agricultural runoff and industrial discharges [4, 6–8]. Such inputs can create hotspots where antimicrobial compounds, ARB and ARGs may co-exist and persist. The presence of antimicrobial compounds and resistance determinants in waters used for recreational purposes raises public health concerns due to potential human exposure and health risks [9, 10].

In this context, surface water and wastewater environments represent critical components of the One Health framework for AMR surveillance, providing insight into anthropogenic drivers and potential dissemination pathways. In Ireland, 48% of surface waters are classified as having moderate, poor or bad ecological status, predominantly due to pollution from agricultural pressures and wastewater discharges [11]. Across Europe, ~60% of surface waters fall below good ecological status, according to assessments by the European Environment Agency (EEA), indicating that ecological pressures on surface waters are widespread, although proportions vary between jurisdictions and reporting cycles [12]. Such pressures may facilitate the introduction and persistence of ARB and ARGs in Irish aquatic environments, highlighting the need for targeted AMR surveillance within national and European surface water monitoring frameworks.

At a European level, the quality of aquatic environments is primarily regulated by the Water Framework Directive (WFD; 2000/60/EC) [13] and the Bathing Water Directive (BWD; 2006/7/EC) [14], although Member States may implement additional national regulations and monitoring programmes to complement these directives. While the BWD monitors faecal indicator organisms, specifically *Escherichia coli* (*E. coli*) and intestinal *Enterococci*, it does not explicitly account for AMR, representing a critical gap in European water quality regulations. Recent revisions to the Urban Wastewater Treatment Directive (UWWTD) (2024/3019) and proposed updates to the WFD aim to harmonize AMR monitoring and allow ARGs to be included in surface water watch lists [15]. These developments reflect the EU’s growing recognition of AMR as both an environmental and public health concern.

To support EU efforts, the European Environment Information and Observation Network Working Group on AMR, assembled by the EEA, conducted a multinational pilot study to assess the feasibility of harmonized AMR monitoring in European surface waters [16]. As part of this initiative, freshwater, seawater and wastewater treatment plant (WWTP) influent and effluent samples from Ireland were analysed for selected ARB and AMR-associated genes. Given the expected influence of wastewater discharges on receiving aquatic environments, it was anticipated that AMR indicators would be highest in wastewater, with reduced abundances in receiving surface waters along the wastewater-to-seawater continuum. The aims of this study were to assess the occurrence and abundance of AMR indicators across key aquatic environments, evaluate the influence of WWTP discharges on receiving surface waters and provide evidence to inform future environmental monitoring.

## Methods

### Overview of sampling sites and collection

**Fig. 1.**
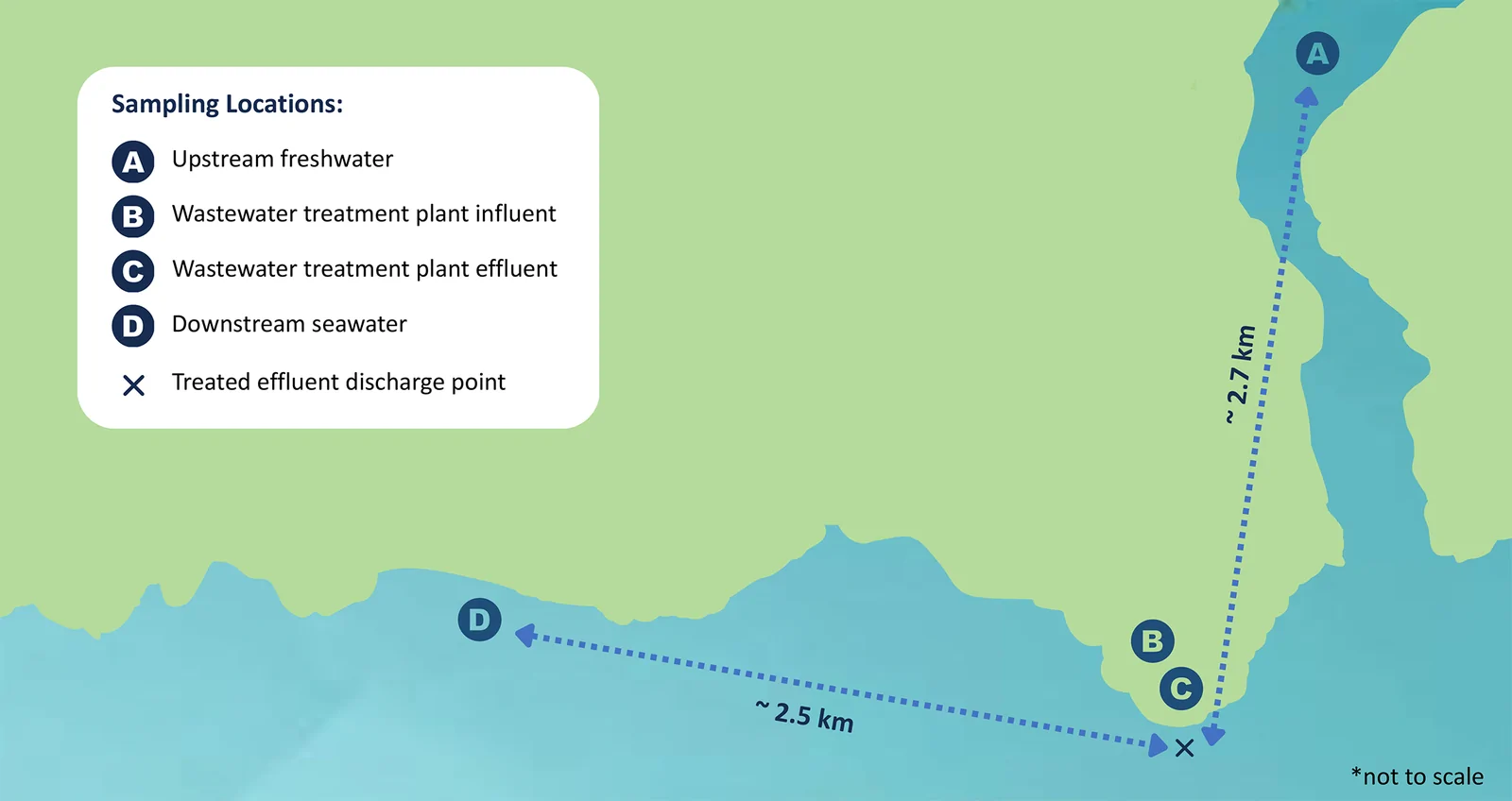
Schematic map of the sampling locations. Approximate distances are shown; the schematic is not to scale and does not represent the actual study location.

Water and wastewater samples were collected on three dates: 12 and 26 November and 3 December 2024. Four sampling sites were selected within a large urban area in Ireland: (1) a freshwater river located upstream of a WWTP, (2) WWTP influent, (3) WWTP effluent and (4) downstream coastal seawater receiving treated effluent (Fig. 1).

The study catchment was selected as it represents a large urban catchment in Ireland, with predominantly municipal wastewater inputs from residential, commercial, healthcare, educational and other institutional sources. The WWTP serves a catchment of ~170,000 population equivalents (p.e.) and operates conventional secondary treatment. Treated effluent is discharged into a coastal water body, which includes a designated bathing area. Sampling locations were selected to characterize AMR along the wastewater–environment continuum, from upstream freshwater, through untreated and treated wastewater, to downstream coastal waters potentially influenced by the WWTP discharge.

Twenty-four-hour composite samples (1 l) of influent and effluent were collected in sterile containers. Upstream freshwater samples were collected following the WHO Tricycle approach [17], whereby three 1 l grab samples were collected ~5 min apart and subsequently pooled together to produce a single 3 l integrated sample for processing. Downstream seawater samples were collected as 3 l grab samples in accordance with EU BWD (2006/7/EC) guidance [14]. All samples were processed within 2–4 h of collection.

### Environmental and meteorological conditions

Physicochemical and environmental parameters were recorded to provide context for microbial and AMR variability. Onsite measurements of water temperature, salinity and pH were obtained using a Hanna Instruments Salinity Tester (HI98319) and pH metre (HI98127) (Hanna Instruments, USA). Environmental metadata were also recorded and included air temperature, wind speed, maximum UV index, humidity and rainfall during the hour prior to sampling and the preceding 24 h, obtained from the Irish National Meteorological Service (Met Éireann). Tidal state for the coastal site was obtained from a publicly available regional tidal prediction service. These metadata were collected to provide contextual information for microbial and AMR patterns but were not used for statistical analysis due to the limited number of sampling events.

### Culture-based analysis

#### Sample preparation and filtration

Freshwater, seawater and wastewater samples were processed for culture-based enumeration of total *E. coli* and presumptive extended-spectrum *β*-lactamase (ESBL)-producing *E. coli*, performed following adaptations of the WHO Global Tricycle protocol [17]. Filtration volumes and dilution factors were selected according to sample type and anticipated bacterial load to obtain countable plates within the quantifiable range (10–100 c.f.u. per plate). Where required, samples were serially diluted tenfold in sterile phosphate‑buffered saline (PBS), and undiluted samples and diluted aliquots were filtered in parallel. The specific filtration volumes and dilution factors applied for total *E. coli* and presumptive ESBL-producing *E. coli* enumeration are provided in Tables S1 and S2 (available in the online Supplementary Material), respectively.

Appropriate sample volumes or dilution aliquots were filtered through 0.45 µm sterile membrane filters using a Microsart^®^ e.jet Laboratory Liquid Transfer Pump (Sartorius, Germany). Duplicate filtrations were performed per sample, and membranes were aseptically transferred onto selective media. One membrane was placed on Tryptone Bile X-glucuronide (TBX) agar (Oxoid, UK) for enumeration of total *E. coli*, and the second on TBX agar supplemented with cefotaxime (4 µg ml^−1^) (Merck, Germany) for detection of presumptive ESBL-producing *E. coli*. Plates were incubated at 37 °C for 18–24 h.

Quality control procedures included negative and positive controls. The negative control consisted of 100 µL sterile PBS filtered through a 0.45 µm sterile membrane filter and plated onto TBX agar. Positive controls included non-ESBL-producing *E. coli* ATCC 25922, ESBL-producing *Klebsiella pneumoniae* ATCC 700603, non-ESBL-producing *K. pneumoniae* ATCC BAA-1706, and an ESBL-producing *E. coli* ST131 laboratory isolate carrying *bla*_CTX-M-15_. Control strains were sub-cultured on TBX agar, TBX supplemented with 4 µg ml^−1^ cefotaxime, and Tryptic Soy Agar (TSA) to verify growth performance and cefotaxime selectivity. Additionally, a quantitative *E. coli* control (NCTC 900 lenticule discs, 30–120 c.f.u.; Merck, Germany) was used to verify filtration accuracy, with the membrane filter placed on TBX agar.

#### Enumeration and confirmation of *E. coli*

Following incubation, presumptive *E. coli* and ESBL-producing *E. coli* colonies were enumerated and expressed as colony forming units per dL (c.f.u. dl^−1^) following correction for dilution and filtered volume. Up to five presumptive ESBL-producing *E. coli* colonies per sample were purified on TBX agar supplemented with cefotaxime (4 µg ml^−1^), followed by a second purification step on TSA.

Species confirmation was performed using matrix-assisted laser desorption/ionisation time-of-flight mass spectrometry (MALDI-TOF MS) in accordance with the manufacturer’s instructions [Bruker; Biotyper Server version 4.1.100 (PYTH)]. All confirmed *E. coli* isolates underwent antimicrobial susceptibility testing using the disc diffusion method in accordance with EUCAST guidelines (EUCAST, v. 14.0). ESBL production was confirmed using cefpodoxime (CPD10; 10 µg) and cefpodoxime–clavulanic acid (CD01; 10/1 µg) combination discs.

### Quantitative real-time PCR analysis

#### Sample preparation and processing

Subsamples were filtered through 0.22 µm sterile membrane filters using a Microsart^®^ e.jet Laboratory Liquid Transfer Pump (Sartorius, Germany). Recommended filtration volumes per membrane varied by sample type and ranged between 250–500 ml for surface waters, 100–200 ml for WWTP effluent and 10–50 ml for WWTP influent. Unless filter clogging occurred, the maximum recommended volumes were filtered for all samples (Table 1). Following filtration, all membranes were aseptically transferred into bead-beating vials, and DNA was extracted from the membrane filters using the DNeasy PowerWater Kit (Qiagen, Germany), as per manufacturer’s instructions. DNA was eluted in a final volume of 100 µl and stored at −20 °C until PCR analysis.

**Table 1.** Volume of samples filtered for quantitative real-time PCR analysis

	12 November	26 November	3 December
**Upstream freshwater**	500 ml	300 ml	500 ml
**WWTP influent**	50 ml	50 ml	50 ml
**WWTP effluent**	200 ml	200 ml	200 ml
**Downstream seawater**	500 ml	500 ml	500 ml

Reduced filtration volume for upstream freshwater on 26 November was due to filter clogging during processing.

#### Detection and quantification of AMR-associated genes

Quantitative real-time PCR was performed on the LightCycler 480 instrument (Roche, Switzerland). Nine previously published PCR assays were adapted for use on this platform, with minor adjustments to cycling parameters and primer concentrations to ensure optimal performance (Table S3). These assays targeted AMR-associated genes *intI1*, *bla*_CTX-M-1_, *vanA*, *ermB*, *aadA1* and the total bacterial load 16S rRNA gene. Targets were selected in accordance with the European pilot study on AMR monitoring in surface waters [16], based on their relevance for environmental AMR surveillance as they represent total bacterial load (16S rRNA), clinically important resistance (*bla*_CTX-M-1_, *vanA*) and commonly observed resistance markers in environmental settings (*ermB*, *aadA1* and *intI1*). A gBlock^®^ DNA was used as a positive control (calibrator), while a negative control (PCR-grade molecular water) and no-template control were included in each real-time PCR run. Reactions were performed using the LightCycler 480 SYBR Green I Master Mix (Roche, Switzerland), followed by melting curve analysis to verify amplification specificity. Detailed sample processing steps, reaction conditions, primers and gBlock specifications are provided in Table S3.

Standard curves were generated for each target to allow for quantification of copies in each sample [18]. A standard gBlock^®^ DNA [Integrated DNA Technologies (IDT), USA] was serially diluted from 4×10^8^ copies μl^-1^ to 4×10^1^ copies μl^-1^ and tested in triplicate in each assay, enabling generation of a standard curve using the LightCycler software. Subsequently, DNA from water and wastewater samples was tested in triplicate for each target, and copy numbers were calculated using the LightCycler software by importing an external standard curve into a run. Copy numbers were expressed as copies per decilitre (copies dl^-1^), in accordance with the pilot study requirements, and summarized using mean values of three technical replicates. No inferential statistical analyses were performed due to the limited number of sampling events.

## Results

### Culture-based detection and abundance of *E. coli* and ESBL-producing *E. coli*

**Fig. 2.**
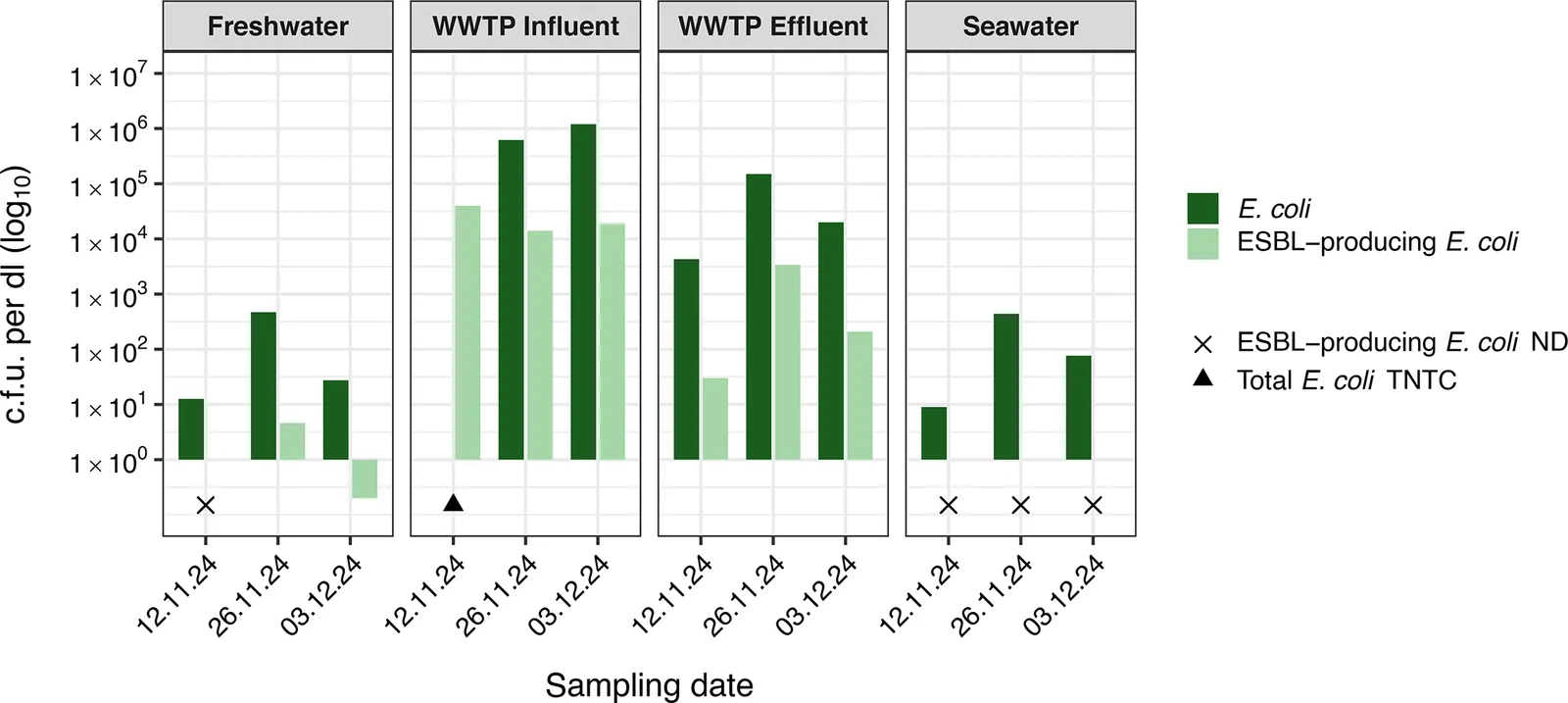
Total *E. coli* and ESBL-producing *E. coli* concentrations in freshwater, WWTP influent, WWTP effluent and seawater samples. Concentrations are expressed as c.f.u. per decilitre (c.f.u. per dl) (log_10_ scale). ND, not detected. Crosses (×) indicate ESBL-producing *E. coli* below the detection limit, and triangles (▲) indicate total *E. coli* TNTC. Sampling dates are shown on the x-axis. This figure was generated in RStudio (version 2025.09.2) using R (version 4.4.1; R Foundation for Statistical Computing, Vienna, Austria) and the ggplot2 package (version 4.0.1).

Total *E. coli* was detected in all samples collected across the sampling period, with concentrations consistently highest in WWTP influent and substantially lower in the surface waters, where the lowest concentrations alternated between downstream seawater and upstream freshwater across sampling dates (Fig. 2). During the first sampling event (12 November), *E. coli* concentrations were 12.6 c.f.u. dl^−1^ in upstream freshwater and 9 c.f.u. dl^−1^ in downstream seawater. ESBL-producing *E. coli* were not detected in either of the surface waters analysed. Total *E. coli* in WWTP influent exceeded the upper limit of quantification, with all plates yielding counts that were too numerous to count (TNTC), preventing accurate enumeration. However, ESBL-producing *E. coli* were detected in WWTP influent at 4×10^4^ c.f.u. dl^−1^. WWTP effluent contained 4.3×10^3^ c.f.u. dl^−1^ total *E. coli* and 30 c.f.u. dl^−1^ ESBL-producing *E. coli*.

*E. coli* was detected in all samples collected during the second sampling event (26 November). Upstream freshwater contained 4.7×10^2^ c.f.u. dl^−1^ total *E. coli*, with 4.6 c.f.u. dl^−1^ ESBL-producing *E. coli*. Downstream seawater had total *E. coli* concentrations of 4.4×10^2^ c.f.u. dl^−1^, while ESBL-producing *E. coli* were not detected. WWTP influent contained 6.2×10^5^ c.f.u. dl^−1^ total *E. coli* and 1.35×10^4^ c.f.u. dl^−1^ ESBL-producing *E. coli*. In WWTP effluent, total *E. coli* concentrations reached 1.5×10^5^ c.f.u. dl^−1^, with ESBL-producing *E. coli* detected at 3.36×10^3^ c.f.u. dl^−1^.

During the final sampling event (3 December), *E. coli* was detected in both upstream freshwater (27.4 c.f.u. dl^−1^) and downstream seawater (76.6 c.f.u. dl^−1^). ESBL-producing *E. coli* were detected at very low abundance in freshwater (<1 c.f.u. dl^−1^), while none were detected in seawater. WWTP influent contained 1.2×10^6^ c.f.u. dl^−1^ total *E. coli* and 1.89×10^4^ c.f.u. dl^−1^ ESBL-producing *E. coli*, while WWTP effluent contained 2.0×10^4^ c.f.u. dl^−1^ total *E. coli* and 2.05×10^2^ c.f.u. dl^−1^ ESBL-producing *E. coli*.

Across all sampling events, a total of 46 presumptive ESBL-producing *E. coli* were recovered. Of these, 43 isolates (93.5%) were confirmed *E. coli*, and 35 (81.4% of confirmed *E. coli*) were phenotypically confirmed as ESBL producers. The confirmed isolates were recovered from freshwater (*n*=3), seawater (*n*=9), WWTP influent (*n*=13) and WWTP effluent (*n*=10), with the highest number recovered on 26 November (*n*=16).

Overall, total *E. coli* concentrations followed a consistent gradient across all sampling events, with the highest levels observed in wastewater influent, followed by WWTP effluent and surface waters. While downstream seawater exhibited the lowest concentrations on two sampling occasions, upstream freshwater had the lowest on the third sampling date. ESBL-producing *E. coli* were predominantly detected in wastewater matrices, particularly influent, while detections in surface waters were sporadic and generally low or below the limit of detection. The highest concentrations of both total and ESBL-producing *E. coli* were recorded on 26 November across WWTP effluent, freshwater and seawater matrices.

### Total bacterial load (16S rRNA gene) abundance

**Fig. 3.**
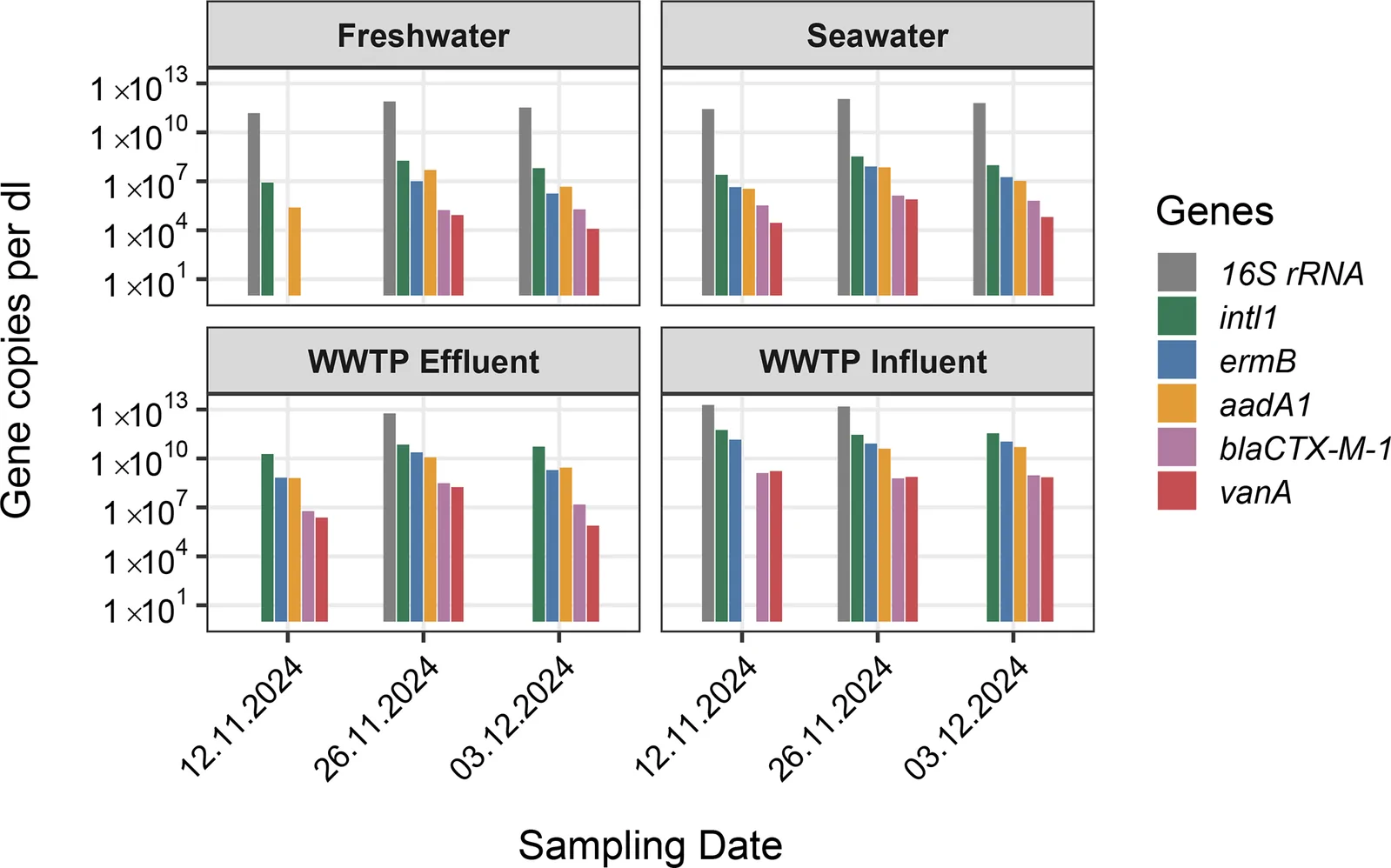
Abundance of the total bacterial marker 16S rRNA and selected AMR-associated genes (*intl1*, *ermB*, *aadA1*, *bla*_CTX-M-1_ and *vanA*) across freshwater, seawater, WWTP effluent and WWTP influent samples collected in 2024. Gene copy numbers are expressed as gene copies per dl (log_10_ scale). Bars represent mean gene copy numbers derived from three real-time PCR replicates. A complete summary of gene copy numbers is provided in Table S4. This figure was generated in RStudio (version 2025.09.2) using R (version 4.4.1; R Foundation for Statistical Computing, Vienna, Austria) and the ggplot2 package (version 4.0.1).

Due to the limited number of sampling dates, results are presented descriptively without statistical analysis. Total bacterial load, quantified by 16S rRNA copy numbers, varied across sampling sites and dates (Fig. 3; Table S4). WWTP influent consistently exhibited the highest abundances, reaching up to 1.9×10^13^ copies dl^-1^. Across sampling dates, influent concentrations were approximately one to two orders of magnitude higher than those observed in surface waters. Downstream seawater copy numbers ranged from 2.68×10^11^ to 1.11×10^12^ copies dl^-1^, while upstream freshwater ranged from 1.5×10^11^ to 7.8×10^11^ copies dl^-1^.

No 16S rRNA amplification was observed in influent on 3 December or in effluent on 12 November and 3 December. As viable bacteria were recovered from these samples (Section 3.1), they were re-tested by real-time PCR to verify the initial results. Non-detection was confirmed, suggesting potential assay inhibition or matrix effects (Section 4.4).

Peak bacterial loads in both freshwater and seawater were recorded on 26 November. Across sampling locations, observed abundances followed the order: influent>effluent>downstream seawater>upstream freshwater.

### Abundance of AMR-associated genes

AMR-associated genes were detected at all sampling sites throughout the duration of the study. Copy numbers varied by several orders of magnitude, with some targets not detected in upstream freshwater on 12 November (Fig. 3; Table S4). WWTP influent consistently exhibited the highest copy numbers for all targets, with *intI1*, *ermB* and *aadA1* reaching up to 5.6×10^11^, 1.4×10^11^ and 5.0×10^10^ copies dl^-1^, respectively. *bla*_CTX-M-1_ and *vanA* were detected at lower levels, generally within the range of 10^8^ to 10^9^ copies dl^-1^.

Although copy numbers were lower in WWTP effluent compared to influent, they were consistently detected and ranged from 10^6^ to 10^10^ copies dl^-1^ depending on the gene and sampling date. Among effluent samples, *intI1*, *ermB* and *aadA1* consistently showed the highest abundances relative to the other targets.

Surface waters contained lower overall copy numbers, ranging from 10^4^ to 10^8^ copies dl^-1^. Downstream seawater copy numbers were generally one to two orders of magnitude higher than upstream freshwater on corresponding dates. Similar to wastewater samples, *intI1*, *ermB* and *aadA1* were the most abundant targets in both surface waters, although *ermB* was not detected in upstream freshwater on 12 November. In addition, *bla*_CTX-M-1_ and *vanA* were not detected on this date.

For most targets, peak abundances were recorded on 26 November in WWTP effluent, upstream freshwater and downstream seawater. Across sampling locations, patterns in AMR gene abundance followed the same spatial gradient observed for total bacterial load (Section 3.2).

## Discussion

### Wastewater as an AMR source and downstream impacts

This pilot provides evidence that wastewater discharge represents an important pathway for the dissemination of AMR determinants into receiving aquatic environments. WWTP influent contained the highest concentrations of total and ESBL-producing *E. coli* as well as the greatest abundance of AMR-associated genes, while treated effluent continued to contain detectable ARB and ARGs. Downstream coastal waters receiving treated effluent exhibited higher abundances of several AMR-associated genes compared with upstream freshwater despite having low concentrations of culturable *E. coli* and the absence of detectable ESBL-producing *E. coli*. These findings indicate that wastewater impacts on environmental AMR can persist beyond treatment and dilution processes and highlight limitations of relying solely on conventional faecal indicator organisms for assessing AMR-related risks in aquatic environments.

While wastewater has long been recognized as an important source of environmental AMR, harmonized monitoring approaches remain under development. This study provides baseline data on the occurrence of ARB and AMR-associated genes across wastewater, freshwater and coastal waters in Ireland, contributing national evidence to ongoing European efforts to evaluate the feasibility of harmonized environmental AMR monitoring.

The combination of culture-based and molecular approaches provided complementary perspectives on environmental AMR dissemination. The 16S rRNA target provided a measure of overall bacterial abundance across different sample types and provided a culture-independent quantitative benchmark for bacterial density [19]. Although 16S rRNA was not consistently detected in all wastewater samples, likely due to matrix-related inhibition (Methodological Considerations and Future Directions), detectable values followed the expected pattern of higher bacterial load in WWTP influent compared with effluent and surface waters, aligning with previous environmental and wastewater studies reporting decreasing bacterial abundance along treatment and dilution gradients [20, 21].

The high concentrations of total and ESBL-producing *E. coli* as well as multiple AMR-associated genes detected in WWTP influent further reinforce the role of wastewater as a major reservoir and pathway for AMR entry into receiving aquatic environments. Across the sampling period, *E. coli* concentrations in WWTP influent ranged from 10^5^ to 10^6^ c.f.u. dl^−1^, with a single sample exceeding the countable range (TNTC), while ESBL-producing *E. coli* were consistently detected at ~10^4^ c.f.u. dl^−1^. All AMR-associated gene targets were detected, with *intI1* and *ermB* exceeding 10^11^ copies dl^−1^. These findings align with previous studies indicating that municipal wastewater harbours substantial concentrations of ESBL-producing *E. coli* and a wide range of AMR-associated genes, supporting the role of raw wastewater as a key source of environmental AMR [4, 22].

In Ireland, as across most of Europe, the majority of municipal wastewater undergoes treatment in WWTPs prior to discharge into the environment; however, conventional treatment processes are not designed to eliminate all ARB and resistance determinants [23]. In this study, although conventional secondary wastewater treatment substantially reduced both culturable *E. coli* and AMR-associated gene abundances, neither was fully eliminated. ESBL-producing *E. coli* remained detectable in effluent (up to ~10^3^ c.f.u. dl^−1^), and all targeted genes persisted at measurable levels, with *intI1*, *ermB* and *aadA1* remaining particularly abundant (~10^10^ copies dl^−1^). The persistence of *E. coli* and AMR-associated genes in treated effluent mirrors patterns observed in other studies. Smyth *et al.* [24] reported high levels of multidrug resistance among faecal coliforms in WWTP effluents in Ireland, reinforcing the persistence of ESBL-producing *E. coli* following treatment. European-wide studies of WWTP effluents by Cacace *et al.* [25] and Pärnänen *et al.* [26] revealed widespread detection of AMR-associated genes in treated effluent, including the class 1 integron marker, *intI1*. Class 1 integrons have been widely recognized as indicators of anthropogenic influence [27], and their dominance in effluent highlights the persistence of mobile genetic elements following conventional treatment. Beyond Europe, metagenomic profiling of internationally sourced wastewater also revealed the persistence of AMR determinants in treated effluents [28], confirming that incomplete removal of AMR determinants is a limitation of conventional wastewater treatment globally.

Downstream seawater contained low concentrations of total *E. coli* and no detectable ESBL-producing *E. coli*, while 16S rRNA and all targeted AMR-associated genes were detected. Compared with upstream freshwater, downstream coastal waters consistently exhibited higher AMR-associated gene copy numbers, suggesting the influence of treated effluent on environmental AMR. For example, *intI1* and *ermB* concentrations reached 3.35×10^8^ and 8.10×10^7^ copies dl^−1^ downstream, compared with 1.83×10^8^ and 1.01×10^7^ copies dl^−1^ upstream. This divergence between culturable indicators and molecular detection suggests that while microbial loads may be reduced, resistance determinants can persist in receiving waters beyond the point of discharge, consistent with previous reports highlighting ARG persistence despite hydrological dilution and natural attenuation processes [29]. The higher ARG abundances observed downstream suggest that treated effluent may contribute to the persistence and transport of resistance determinants within the wastewater-to-receiving water continuum, although additional environmental sources cannot be excluded. The absence of culturable ESBL-producing *E. coli* downstream does not necessarily indicate the absence of wastewater-derived resistance determinants, as quantitative polymerase chain reaction (qPCR)-based detection can detect both intracellular and extracellular DNA and may detect ARGs associated with non-culturable or non-*E. coli* bacterial populations [30].

Of particular concern was the detection of clinically relevant ARGs, especially *bla*_CTX-M-1_, in downstream recreational waters. Although detected at lower abundances than in wastewater, their presence remains concerning given their association with resistance to clinically important antimicrobials. These findings are consistent with observations from a previous Irish study documenting detectable levels of clinically relevant ARGs in coastal waters influenced by WWTP discharge [4]. Similar persistence of clinically significant AMR-associated genes downstream of WWTPs has also been reported by Knight *et al.* [31], who detected multiple ARGs, including *bla*_CTX-M_, in coastal waters despite observing a decline in culturable bacterial indicators, also consistent with the pattern observed in this study. Although, in this study, copy numbers in surface waters were lower than those in wastewater, the presence of clinically significant AMR-associated genes may still contribute to potential exposure risks, supporting the need for enhanced surveillance and improved wastewater management.

### Temporal patterns in AMR indicators and environmental conditions

Temporal variability in microbial indicator concentrations and AMR-associated gene copy numbers was observed across all sample types, with peak values recorded in WWTP effluent and surface waters on 26 November. This sampling event followed a period of heavy rainfall and storm conditions, during which up to 44.5 mm of rainfall was recorded within a 24 h period. Extreme weather events are known to influence microbial and ARG loads in aquatic environments through increased wastewater flows, reduced treatment efficiency, combined sewer overflows, stormwater runoff and resuspension of contaminated sediments [32–34]. However, the limited number of sampling events in this study precluded assessment of a statistical association between rainfall and ARG abundance.

Although elevated ARG copy numbers were observed during the sampling event following heavy rainfall, these findings represent temporal observations rather than evidence of a causal relationship. The limited sampling period and absence of inferential statistical analyses preclude attribution of the observed increases to rainfall. However, the observed temporal pattern is consistent with previous studies reporting elevated ARG loads following wet weather events. Stormwater runoff has been identified previously as a key pathway for AMR dissemination, with heavy rainfall mobilizing ARB, ARGs and antibiotic residues from land sources into receiving waters, contributing to temporal fluctuations in resistome profiles [35, 36]. Similarly, elevated ARG copy numbers have been reported in surface waters following wet weather events, suggesting that rainfall may enhance ARG transport even when dilution occurs [37, 38]. As real-time PCR detects both viable and non-viable DNA, extracellular DNA released or transported during such events may also contribute to increased AMR signals. This aligns with evidence that extracellular DNA can persist in aquatic environments and potentially serve as a mobile resistance gene reservoir [39]. These observations highlight the need for larger longitudinal studies incorporating formal statistical analyses to evaluate the influence of meteorological conditions on environmental AMR.

### Implications for environmental surveillance and EU water policy

Detection of AMR-associated genes in surface waters impacted by wastewater discharges suggests that conventional faecal indicator-based monitoring may underestimate emerging AMR-related risks. Although current EU water quality frameworks effectively assess general microbial contamination, the persistence of resistance determinants downstream of treated effluent highlights a gap in their capacity to capture anthropogenic AMR pressures [26]. Evidence has shown that, despite compliance with regulatory standards for faecal indicator bacteria (FIB), clinically significant ARB and ARGs can remain abundant in wastewater-impacted surface waters indicating that traditional indicators may not capture all relevant AMR-related hazards [26, 40]. Correlations between ARGs and FIB have been observed under continuous wastewater influence; however, they are often inconsistent in more complex or intermittently impacted environments [41]. Consistent with previous studies, the coastal waters investigated in this study would be classified as ‘excellent’ or ‘good’ based on FIB, under the EU BWD, despite the presence of clinically significant ARGs (2006/7/EC) [14, 40]. These results reinforce the need for enhanced surveillance strategies incorporating resistance markers into coastal water risk assessments to better capture AMR-related hazards [3].

Recent policy developments reflect the growing recognition of environmental AMR data within a One Health framework and align with broader efforts to strengthen water quality legislation and monitoring in the EU. The revised UWWTD (2024/3019) mandates AMR monitoring in urban wastewater from larger agglomerations (>100,000 p.e.) by 2030, while proposed revisions to the WFD include provisions for the inclusion of AMR indicators on surface water watch lists [42, 43]. The findings of this study support these initiatives by demonstrating that AMR-associated genes and resistant bacteria can be reliably detected in surface waters using standardized methodologies and that wastewater discharges represent a pathway for environmental AMR dissemination.

The consistent detection of *intI1* across freshwater, wastewater and coastal environments supports its potential use as a marker of anthropogenic AMR pollution [27, 44], while the presence of clinically significant ARGs highlights the relevance of targeted gene panels for environmental surveillance [45]. These results demonstrate the value of integrating AMR surveillance into existing water quality monitoring programmes and support the inclusion of resistance indicators in future EU regulatory frameworks. In Ireland, this aligns with priorities outlined in the One Health National Action Plan on Antimicrobial Resistance (2026–2030), which emphasizes the inclusion of environmental surveillance as part of a coordinated national response to AMR [46].

### Methodological considerations and future directions

Effective environmental AMR surveillance requires careful methodological and interpretive consideration. In this study, the combination of culture-based and molecular approaches provided complementary perspectives on environmental AMR. Culture-based analyses allow for confirmation of phenotypic resistance, organism viability and information relevant to potential exposure risks, while real-time PCR enables sensitive detection and quantification of resistance genes across diverse environmental samples. Differences between the two approaches, such as the intermittent detection of ESBL-producing *E. coli* by culture and consistent detection of AMR-associated genes by real-time PCR, likely reflect the presence of viable but non-culturable bacteria, extracellular DNA and resistance determinants harboured by non-*E. coli* taxa, as well as differences in analytical sensitivity [30, 47]. These findings reinforce the importance of integrating phenotypic and genotypic methods in surveillance programmes to provide a comprehensive assessment of environmental AMR occurrence and potential risk [48]. Such integration may also support the identification of AMR indicators suitable for inclusion in future EU surface water watch lists.

Several limitations should be considered when interpreting PCR-based results. Real-time PCR cannot distinguish between viable and non-viable genetic material, potentially leading to overestimation of exposure risks. In addition, PCR performance may be influenced by matrix-specific inhibition. In this study, the 16S rRNA gene, used as a marker of total bacterial load, was not detected in some wastewater samples despite high culturable bacterial counts and detectable AMR-associated genes, suggesting that PCR inhibition or matrix-specific extraction challenges, rather than the true absence of bacterial DNA, may have contributed to these findings. Such effects are well recognized in complex environmental matrices, particularly wastewater, where organic matter and inhibitory substances can interfere with amplification efficiency [49, 50]. Similar considerations apply to marine environments. High salinity and osmotic stress may promote the transition of *E. coli* into a viable but non-culturable state [51], which may explain the absence of recoverable ESBL-producing *E. coli* despite the detection of ARGs by qPCR in this study. Recent comparative studies further demonstrate that molecular approaches used for environmental AMR surveillance vary in sensitivity depending on the method applied, reinforcing the need for careful interpretation and robust quality control when generating quantitative molecular AMR data from complex matrices [52].

The targeted nature of the AMR-associated gene panel used in this pilot study should also be acknowledged. The selected targets were aligned with the European pilot initiative and represented key clinically and environmentally relevant AMR indicators. However, this targeted approach provides only a partial assessment of the environmental resistome and does not capture other resistance mechanisms or broader ARG diversity. Therefore, resistance determinants that may be relevant in specific environmental settings, but are not in the selected panel, would not have been detected.

The generalizability of these findings should also be considered in the context of the pilot study design. Sampling was conducted within a single urban catchment over three sampling occasions in late 2024, and therefore, the observed AMR patterns may not capture the full spatial and seasonal variability of wastewater-impacted aquatic environments across Europe. As this study formed part of a European pilot initiative for harmonized AMR monitoring, its primary focus was to evaluate the feasibility of implementing standardized AMR monitoring approaches and assess the occurrence of selected AMR indicators across wastewater and receiving water environments. Further harmonized surveillance across diverse geographical settings and longer monitoring periods is required to better characterize regional variability and establish broader environmental AMR trends at a European scale.

Future studies could consider extending monitoring periods and spatial coverage to better characterize temporal and spatial variability, including seasonal trends and comparative assessment of different WWTP processes. Incorporating metagenomic approaches could also improve characterization of ARG diversity, host associations and mobility [47, 52, 53]. Integration of meteorological and hydrological data could also enhance understanding of the drivers influencing AMR variability, inform risk assessment and support evidence-based policy development. Despite these considerations, consistent patterns across samples, dates and analytical approaches support the robustness of these pilot study findings. Although conducted within a single catchment, the harmonized methodology and observed spatial patterns demonstrate the value of environmental AMR surveillance for informing risk assessment and guiding future monitoring across wastewater-impacted aquatic environments in Europe.

## Conclusion

This pilot study indicates that municipal wastewater can be a significant source of AMR determinants in surface waters. Conventional secondary wastewater treatment substantially reduces bacterial loads and ARG abundance but does not fully eliminate ESBL-producing *E. coli* or clinically relevant resistance genes. Detection of ARGs downstream of treated effluent, despite low culturable indicators, highlights limitations of faecal indicator-based monitoring and supports the need for combined phenotypic and genotypic surveillance.

Temporal variability was observed across sampling events, with elevated ARG copy numbers following heavy rainfall. Although the limited dataset precludes inference regarding weather-related drivers, the observed pattern is consistent with previous reports and warrants investigation in larger longitudinal studies. These findings support the inclusion of resistance markers in EU water monitoring frameworks and highlight the need for expanded harmonized environmental surveillance. Future work should broaden spatial and temporal sampling, evaluate additional treatment technologies and incorporate metagenomic approaches to better characterize ARG diversity, mobility and public health relevance.

## Supplementary material

10.1099/mic.0.001771 Supplementary Material 1.

## Abbreviations

AMR: antimicrobial resistance
ARB: antimicrobial-resistant bacteria
ARGs: antimicrobial-resistant bacteria and genes
BWD: Bathing Water Directive
c.f.u.dl−1: colony forming units per decilitre
copies dl-1: copies per decilitre
E. coli: Escherichia coli
EEA: European Environment Agency
ESBL: extended-spectrum *β*-lactamase
FIB: faecal indicator bacteria
MALDI-TOF MS: matrix-assisted laser desorption/ionisation time-of-flight mass spectrometry
PBS: phosphate‑buffered saline
p.e.: population equivalents
qPCR: quantitative polymerase chain reaction
TBX: Tryptone Bile X-glucuronide
TNTC: too numerous to count
TSA: Tryptic Soy Agar
UWWTD: Urban Wastewater Treatment Directive
WFD: Water Framework Directive
WHO: World Health Organization
WWTP: wastewater treatment plant
