# Supplementary material for: Antimicrobial resistance in wastewater-impacted coastal waters: implications for environmental surveillance and public health
Source: Microbiology (Reading). 2026 Sep 7;172(9):001771. doi: 10.1099/mic.0.001771 (PMC13549477; doi:10.1099/mic.0.001771)
Supplement: Supplementary file 1 [file mic-172-01771-s001.pdf]

SUPPORTING INFORMATION

For

Antimicrobial Resistance in Wastewater-Impacted Coastal Waters: Implications for Environmental Surveillance and Public Health

Niamh Cahill[a,b], Aneta Kovarova[a,b], Zina Alfahl[a,b], Louise O'Connor[a,b,c], Dearbháile Morris[a,b].

[a]Antimicrobial and Microbial Ecology Group, School of Medicine, University of Galway, Ireland

[b]Centre for One Health, Ryan Institute, University of Galway, Ireland

[c]Molecular Diagnostics Research Group, School of Biological and Chemical Sciences, University of Galway, Ireland.

Corresponding Author: Niamh Cahill; email: niamh.cahill@universityofgalway.ie; postal address: Discipline of Bacteriology, Orbsen Building, University of Galway, Galway, Ireland.

Supplementary Appendix

Supplementary Table S1: Filtration volumes and dilution factors for total *E. coli* enumeration.

Sampling Site	Sampling Date	Dilution	Volume filtered (mL)
**Upstream Freshwater**	12th November 2024	Undiluted	100
		Undiluted	10
		Undiluted	1
		10^{-1}	1
**Upstream Freshwater**	26th November 2024	Undiluted	250
		Undiluted	100
		Undiluted	10
		Undiluted	1
**Upstream Freshwater**	3rd December 2024	Undiluted	250
		Undiluted	100
		Undiluted	10
		Undiluted	1
**WWTP Influent**	12th November 2024	Undiluted	100
		Undiluted	10
		Undiluted	1
		10^{-1}	1
**WWTP Influent**	26th November 2024	Undiluted	1
		10^{-1}	1
		10^{-2}	1

		10^{-3}	1
**WWTP Influent**	3rd December 2024	Undiluted	1
		10^{-1}	1
		10^{-2}	1
		10^{-3}	1
**WWTP Effluent**	12th November 2024	Undiluted	100
		Undiluted	10
		Undiluted	1
		10^{-1}	1
**WWTP Effluent**	26th November 2024	Undiluted	10
		Undiluted	1
		10^{-1}	1
		10^{-2}	1
**WWTP Effluent**	3rd December 2024	Undiluted	10
		Undiluted	1
		10^{-1}	1
		10^{-2}	1
**Downstream Seawater**	12th November 2024	Undiluted	10
		Undiluted	1
		10^{-1}	1
		10^{-2}	1
**Downstream Seawater**	26th November 2024	Undiluted	250
		Undiluted	100

		Undiluted	10
		Undiluted	1
**Downstream Seawater**	3rd December 2024	Undiluted	250
		Undiluted	100
		Undiluted	10
		Undiluted	1

Notes: Undiluted samples are indicated as 'Undiluted'; dilution factors represent ten-fold serial dilutions prepared in sterile PBS.

Supplementary Table S2: Filtration volumes and dilution factors for presumptive ESBL-producing *E. coli* enumeration.

Sampling Site	Sampling Date	Dilution	Volume filtered (mL)
**Upstream Freshwater**	12th November 2024	Undiluted	100
		Undiluted	100
		Undiluted	100
		Undiluted	10
**Upstream Freshwater**	26th November 2024	Undiluted	250
		Undiluted	100
		Undiluted	10
		Undiluted	1
**Upstream Freshwater**	3rd December 2024	Undiluted	250
		Undiluted	100
		Undiluted	50
		Undiluted	10
**WWTP Influent**	12th November 2024	Undiluted	10
		Undiluted	1
		10^{-1}	1
		10^{-2}	1
**WWTP Influent**	26th November 2024	Undiluted	1
		10^{-1}	1
		10^{-2}	1
		10^{-3}	1

**WWTP Influent**	3rd December 2024	Undiluted	1
		10^{-1}	1
		10^{-2}	1
		10^{-3}	1
**WWTP Effluent**	12th November 2024	Undiluted	10
		Undiluted	1
		10^{-1}	1
		10^{-2}	1
**WWTP Effluent**	26th November 2024	Undiluted	100
		Undiluted	10
		Undiluted	1
		10^{-1}	1
**WWTP Effluent**	3rd December 2024	Undiluted	100
		Undiluted	10
		Undiluted	1
		10^{-1}	1
**Downstream Seawater**	12th November 2024	Undiluted	100
		Undiluted	100
		Undiluted	100
		Undiluted	10
**Downstream Seawater**	26th November 2024	Undiluted	250
		Undiluted	100
		Undiluted	10

		Undiluted	1
**Downstream Seawater**	3rd December 2024	Undiluted	250
		Undiluted	100
		Undiluted	50
		Undiluted	10

Notes: Undiluted samples are indicated as 'Undiluted'; dilution factors represent ten-fold serial dilutions prepared in sterile PBS.

Supplementary Table S3. qPCR assays for AMR resistance genes

Target	Primer name	Primer sequence	Amplicon size (bp)	Ta ºC	Cycling / primer concentration	Reference
**16S rRNA**	P1 / 341F	CCTACGGGAGGCAGCAG	193-194	60	Two steps with 10s at 95^{0}C + 20s at 60^{0}C / 0.3 μM	(Muyzer, de Waal et al. 1993, Uyaguari-Diaz, Chan et al. 2016, Sandberg, Ishii et al. 2018)
	P2 / 518R	ATTACCGCGGCTGCTGG				
intI1 **Class 1 integron integrase**	intI1-LC1	GCCTTGATGTTACCCGAGAG	196	60	Two steps with 10s at 95^{0}C + 20s at 60^{0}C / 0.6 μM	(Barraud, Baclet et al. 2010, Iakovides, Michael-Kordatou et al. 2019)
	intI1-LC5	GATCGGTCGAATGCGTGT				
***bla*$_{CTX-M1}$**	RTCTXM-F	CTATGGCACCACCAACGATA	103	60	Two steps with 10s at 95^{0}C + 20s at 60^{0}C / 0.3 μM	(Marti, Jofre et al. 2013, Herrig, Fleischmann et al. 2020, Lee, Ju et al. 2021)
	RTCTXM-R	ACGGCTTTCTGCCTTAGGTT				
vanA	vanAF	CATGGCAAGTCAGGTGAAGA	187	58	Two steps with 10s at 95^{0}C + 30s at 58^{0}C / 0.2 μM	(Subirats, Triadó-Margarit et al. 2017)
	vanAR	CCACCGGCCTATCATCTTT				
ermB	ermB-F	TGAATCGAGACTTGAGTGTGCAA	71	60	Two steps with 10s at 95^{0}C + 30s at 60^{0}C / 0.2 μM	(Alexander, Bollmann et al. 2015, Hembach, Schmid et al. 2017)
	ermB-R	GGATTCTACAAGCGTACCTT				
aadA1	aadA-F	GTTGTGCACGACGACATCATT	101	60	Two steps with 10s at 95^{0}C + 60s at 60^{0}C / 0.4 μM	(Zhu, Johnson et al. 2013, Iakovides, Michael-Kordatou et al. 2019)
	aadA-R	GGCTCGAAGATACCTGCAAGAA				

gBlock (IDT, Europe):

TttttCCTACGGGAGGCAGCAGTGGGGAATATTGCACAATGGGCGCAAGCCTGATGCAGCCATGCCGCGTGTaTGAAGAAGGCCTTCGGGTTGTAA
AGtACTTTCAGcgggGAGGAAGGgagTgaagTTAATAccttcacTcaTTGACGTTAcccgCAGAAGAAGCACCGGCTAACTCCGTGCCAGCAGCCGCGGTAAT
tttttGCCTTGATGTTACCCGAGAGCTTGGCACCCAGCCTGCGCGAGCAGCTGTCGCGTGCACGGGCATGGTGGCTGAAGGACCAGGCCGAGGGCCG
CAGCGGCGTTGCGCTTCCCGACGCCCTTGAGCGGAAGTATCCGCGCGCCGGGCATTCCTGGCCGTGGTTCTGGGTTTTTGCGCAGCACACGCATT
CGACCGATCtttttCTATGGCACCACCAACGATATCGCGGTGATCTGGCCAAAAGATCGTGCGCCGCTGATTCTGGTCACTTACTTCACCCAGCCTCA
ACCTAAGGCAGAAAGCCGTtttttCATGGCAAGTCAGGTGAAGATGGATCCATACAAGGTCTGTTTGAATTGTCCGGTATCCCTTTTGTAGGCTGCG
ATATTCAAAGCTCAGCAATTTGTATGGACAAATCGTTGACATACATCGTTGCGAAAAATGCTGGGATAGCTACTCCCGCCTTTTGGGTTATTAAT
AAAGATGATAGGCCGGTGGtttttTGAATCGAGACTTGAGTGTGCAAGAGCAACCCTAGTGTTCGGTGAATATCCAAGGTACGCTTGTAGAATCCttttt
GTTGTGCACGACGACATCATTCCGTGGCGTTATCCAGCTAAGCGCGAACTGCAATTTGGAGAATGGCAGCGCAATGACATTCTTGCAGGTATCTT
CGAGCCttttt

REFERENCES

Alexander, J., A. Bollmann, W. Seitz and T. Schwartz (2015). "Microbiological characterization of aquatic microbiomes targeting taxonomical marker genes and antibiotic resistance genes of opportunistic bacteria." Science of The Total Environment **512-513**: 316-325.

Barraud, O., M. C. Baclet, F. Denis and M. C. Ploy (2010). "Quantitative multiplex real-time PCR for detecting class 1, 2 and 3 integrons." Journal of Antimicrobial Chemotherapy **65**(8): 1642-1645.

Hembach, N., F. Schmid, J. Alexander, C. Hiller, E. T. Rogall and T. Schwartz (2017). "Occurrence of the mcr-1 Colistin Resistance Gene and other Clinically Relevant Antibiotic Resistance Genes in Microbial Populations at Different Municipal Wastewater Treatment Plants in Germany." Front Microbiol **8**: 1282.

Herrig, I., S. Fleischmann, J. Regnery, J. Wesp, G. Reifferscheid and W. Manz (2020). "Prevalence and seasonal dynamics of blaCTX-M antibiotic resistance genes and fecal indicator organisms in the lower Lahn River, Germany." PLoS One **15**(4): e0232289.

Iakovides, I. C., I. Michael-Kordatou, N. F. F. Moreira, A. R. Ribeiro, T. Fernandes, M. F. R. Pereira, O. C. Nunes, C. M. Manaia, A. M. T. Silva and D. Fatta-Kassinos (2019). "Continuous ozonation of urban wastewater: Removal of antibiotics, antibiotic-resistant Escherichia coli and antibiotic resistance genes and phytotoxicity." Water Research **159**: 333-347.

Lee, J., F. Ju, A. Maile-Moskowitz, K. Beck, A. Maccagnan, C. S. McArdell, M. Dal Molin, F. Fenicia, P. J. Vikesland, A. Pruden, C. Stamm and H. Bürgmann (2021). "Unraveling the riverine antibiotic resistome: The downstream fate of anthropogenic inputs." Water Research **197**: 117050.

Marti, E., J. Jofre and J. L. Balcazar (2013). "Prevalence of Antibiotic Resistance Genes and Bacterial Community Composition in a River Influenced by a Wastewater Treatment Plant." PLOS ONE **8**(10): e78906.

Muyzer, G., E. C. de Waal and A. G. Uitterlinden (1993). "Profiling of complex microbial populations by denaturing gradient gel electrophoresis analysis of polymerase chain reaction-amplified genes coding for 16S rRNA." Appl.Environ.Microbiol. **59**(3): 695-700.

Sandberg, K. D., S. Ishii and T. M. LaPara (2018). "A Microfluidic Quantitative Polymerase Chain Reaction Method for the Simultaneous Analysis of Dozens of Antibiotic Resistance and Heavy Metal Resistance Genes." Environmental Science & Technology Letters **5**(1): 20-25.

Subirats, J., X. Triadó-Margarit, L. Mandaric, V. Acuña, J. L. Balcázar, S. Sabater and C. M. Borrego (2017). "Wastewater pollution differently affects the antibiotic resistance gene pool and biofilm bacterial communities across streambed compartments." Molecular Ecology **26**(20): 5567-5581.

Zhu, Y.-G., T. A. Johnson, J.-Q. Su, M. Qiao, G.-X. Guo, R. D. Stedtfeld, S. A. Hashsham and J. M. Tiedje (2013). "Diverse and abundant antibiotic resistance genes in Chinese swine farms." Proceedings of the National Academy of Sciences **110**(9): 3435-3440.

Supplementary Table S4: Abundance of total bacterial load and selected AMR-associated genes across freshwater, WWTP influent, WWTP effluent and seawater samples collected in 2024

Sampling Site	Sampling Date	Target Genes (copies/dL)					
		**16S rRNA**	***intI1***	***ermB***	***aadA1***	***bla*$_{CTX-M-1}$**	***vanA***
Upstream Freshwater	12th November 2024	$1.5×10^{11}$	$8.4×10^{6}$	ND	$2.5×10^{5}$	ND	ND
Upstream Freshwater	26th November 2024	$7.8×10^{11}$	$1.8×10^{8}$	$1.0×10^{7}$	$5.0×10^{7}$	$1.7×10^{5}$	$8.6×10^{4}$
Upstream Freshwater	3rd December 2024	$3.3×10^{11}$	$6.4×10^{7}$	$1.8×10^{6}$	$4.6×10^{6}$	$1.9×10^{5}$	$1.2×10^{4}$
WWTP Influent	12th November 2024	$1.9×10^{13}$	$5.6×10^{11}$	$1.4×10^{11}$	ND	$1.3×10^{9}$	$1.7×10^{9}$
WWTP Influent	26th November 2024	$1.5×10^{13}$	$2.8×10^{11}$	$8.3×10^{10}$	$4.0×10^{10}$	$6.0×10^{8}$	$7.4×10^{8}$
WWTP Influent	3rd December 2024	ND	$3.5×10^{11}$	$1.1×10^{11}$	$5.0×10^{10}$	$9.2×10^{8}$	$7.0×10^{8}$
WWTP Effluent	12th November 2024	ND	$1.9×10^{10}$	$6.7×10^{8}$	$6.3×10^{8}$	$5.9×10^{6}$	$2.4×10^{6}$
WWTP Effluent	26th November 2024	$5.8×10^{12}$	$7.1×10^{10}$	$2.4×10^{10}$	$1.2×10^{10}$	$3.1×10^{8}$	$1.8×10^{8}$
WWTP Effluent	3rd December 2024	ND	$5.4×10^{10}$	$2.0×10^{9}$	$2.8×10^{9}$	$1.5×10^{7}$	$7.6×10^{5}$
Downstream Seawater	12th November 2024	$2.7×10^{11}$	$2.5×10^{7}$	$4.4×10^{6}$	$3.5×10^{6}$	$3.3×10^{5}$	$2.9×10^{4}$

Downstream Seawater	26th November 2024	1.1×10^{12}	3.4×10^{8}	8.1×10^{7}	7.1×10^{7}	1.3×10^{6}	7.8×10^{5}
Downstream Seawater	3rd December 2024	6.2×10^{11}	9.7×10^{7}	1.8×10^{7}	1.1×10^{7}	6.4×10^{5}	6.5×10^{4}

Note: ND = Not Detected. Copy numbers are expressed as copies per decilitre (copies/dL). Values represent mean copy numbers derived from three real-time PCR replicates.
